# Prognostic nutritional index in evaluating the prognosis of ICIs in patients with advanced esophageal squamous carcinoma

**DOI:** 10.3389/fonc.2026.1764557

**Published:** 2026-08-24

**Authors:** Qingfang Shi, Man Li, Daqing Wang, Jun’e Yang, Jun Gao

**Affiliations:** Department of Medical Oncology, Hengshui People’s Hospital, Hengshui, China

**Keywords:** esophageal squamous cell carcinoma, immune checkpoint inhibitors, prognosis, prognostic nutritional index, stratification marker

## Abstract

**Objective:**

This study aimed to investigate the association between the Prognostic Nutritional Index (PNI) and the prognosis of advanced esophageal squamous cell carcinoma (ESCC) patients treated with immune checkpoint inhibitors (ICIs).

**Methods:**

We retrospectively analyzed the clinical data of 157 advanced ESCC patients who received ICIs therapy at Hengshui People’s Hospital. The PNI was calculated based on serum albumin levels and lymphocyte counts. The predictive value of PNI for disease progression in advanced ESCC patients was evaluated using receiver operating characteristic (ROC) curve analysis.

**Results:**

Among 157 patients with advanced ESCC, the complete response (CR) rate was 0.64% (1/157), partial response (PR) rate was 47.13% (74/157), stable disease (SD) rate was 38.22% (60/157), and progressive disease (PD) rate was 14.01% (22/157). The median progression-free survival (PFS) was 14.2 months. During the follow-up period, 80 patients died, leading to a mortality rate of 50.96%. The CR+PR group showed significantly higher albumin levels, lymphocyte counts, and PNI compared to the PD group (*p*< 0.05). The area under the curve (AUC) of PNI for predicting disease progression was 0.770 (95% CI: 0.696-0.833), with a sensitivity of 86.36% and specificity of 58.52%. Based on the cutoff value, patients were stratified into high-PNI (>44.4, n=82) and low-PNI (ow-PNI n=75) groups. The high-PNI group demonstrated significantly lower disease progression rates compared to the low-PNI group (*p*< 0.05). Kaplan-Meier analysis revealed that advanced ESCC patients in the high-PNI group had significantly longer PFS and overall survival (OS) than those in the low-PNI group (*p*< 0.05). The disease-free survival rate after ICIs therapy was 96.34% in the high-PNI group compared to 74.67% in the low-PNI group (Log-rank χ²=18.332, *p*< 0.05). The 3-year survival rate post-ICIs was 64.93% in the high-PNI group compared to 33.75% in the low-PNI group (Log-rank χ²=12.378, *p*< 0.05). Lymphocyte count and PNI as independent prognostic factors for PFS in advanced ESCC patients (*p*< 0.05), while poor differentiation, smoking history, and PNI were independent predictors for OS (*p*< 0.05).

**Conclusion:**

PNI is associated with the efficacy of ICI therapy and prognosis in patients with advanced ESCC, suggesting its potential as a promising prognostic stratification marker. However, the clinical utility of PNI as an independent biomarker requires further validation through prospective, multicenter studies with larger sample sizes.

## 1 Introduction

Esophageal squamous cell carcinoma (ESCC), the predominant histological subtype of esophageal cancer, exhibits persistently high incidence and mortality rates in China, with a 5-year survival rate below 20% for advanced-stage patients (1). Recent advances with immune checkpoint inhibitors (ICIs), including pembrolizumab and nivolumab, have significantly improved survival outcomes in advanced ESCC. However, substantial interindividual variability in treatment response remains a clinical challenge (2). Accurate prognostic stratification is therefore critical for advanced ESCC management. Current predictive biomarkers primarily focus on tumor molecular characteristics or clinicopathological parameters (e.g., PD-L1 expression, TNM staging) (3, 4). While these indicators provide some prognostic value, their inability to reflect systemic immune status and treatment tolerance limits clinical utility. Nutritional assessment plays an indispensable role in advanced ESCC treated with ICIs, as malnutrition directly suppresses T-cell proliferation, reduces cytokine secretion, and consequently attenuates anti-tumor immune responses. The prognostic nutritional index (PNI), which integrates peripheral lymphocyte counts and serum albumin levels, has demonstrated predictive potential for chemoradiotherapy efficacy and survival outcomes in various solid tumors, including gastric and lung cancers (5, 6). A previous study indicates that PNI indirectly evaluates treatment tolerance and tumor immune microenvironment activity by reflecting systemic immune reserves and metabolic status (7). Notably, 60%-80% of ESCC patients present with malnutrition, which not only accelerates cachexia progression but may also impair the efficacy of ICIs through T-cell dysfunction and reduced sensitivity to immunotherapy (8). However, the prognostic value of the PNI in patients with advanced ESCC receiving ICIs therapy remains unclear, and there is a particular lack of evidence supporting individualized immunotherapy based on nutritional-immunity integrated indicators. This retrospective study aims to investigate the association between PNI and survival outcomes in advanced ESCC patients receiving ICIs, evaluating its potential as a prognostic biomarker. This study may provide several insights for the clinical management of patients with ESCC: early nutritional intervention and close monitoring of disease progression are recommended for patients with a low PNI, whereas those with a high PNI may be prioritized for immunotherapy, thereby facilitating stratified management and precision medicine.

## 2 Materials and methods

### 2.1 Patients

**Figure 1:**
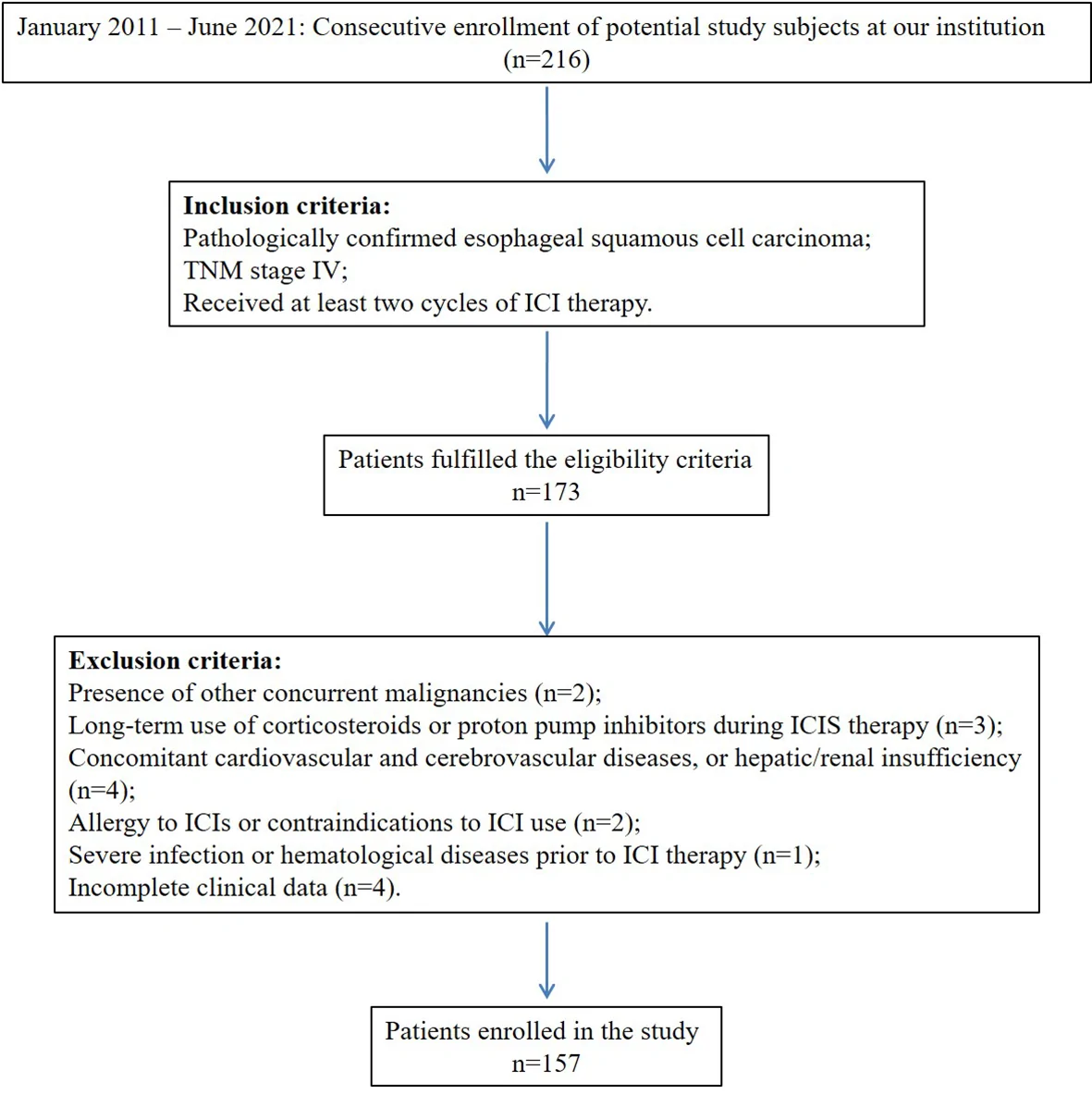
Patient selection flowchart.

This retrospective study enrolled 157 patients with advanced ESCC who were diagnosed at Hengshui People’s Hospital between January 2011 and June 2021. Inclusion criteria (**Table 1**): (1) pathologically confirmed ESCC; (2) TNM stage IV; (3) received at least two cycles of ICIs therapy. Exclusion criteria (**Table 1**): (1) concurrent other malignancies; (2) incomplete clinical data; (3) long-term use of corticosteroids or proton pump inhibitors during ICIs treatment; (4) active severe infection or hematologic disorders prior to ICIs therapy; (5) known hypersensitivity or contraindications to ICIs; (6) comorbid severe cardiovascular, cerebrovascular, hepatic, or renal diseases. The detailed inclusion and exclusion criteria were presented in **Table 1**, and the patient selection flowchart was shown in **Figure 1**.The study protocol was approved by the Institutional Review Board/Ethics Committee of Hengshui People’s Hospital. All patients provided informed consent to participate in this study.

**Table 1:** Inclusion and exclusion criteria for study subjects.

Categories	Specific criteria	Definition/following instructions
Inclusion Criteria	Pathological diagnosis was ESCC	Squamous cell carcinoma was confirmed by endoscopic biopsy and histopathology of surgical resection specimens
	TNM staging was stage IV	According to the AJCC/UICC 8th edition staging system, patients had distant metastasis or unresectable locally advanced disease
	Patients received at least 2 cycles of ICIs	Patients received PD-1/PD-L1 inhibitor treatment for ≥2 consecutive cycles to ensure that drug exposure could be evaluated
Exclusion Criteria	Combined with other malignant tumors	Previous or current history of other primary malignancies other than ESCC
	The clinical data were incomplete	Baseline nutritional inflammation measures, treatment information, or key items of survival follow-up data were missing
	Long-term use of hormone/proton pump inhibitors	Regular oral administration of corticosteroids or proton pump inhibitors for ≥2 weeks during ICIs treatment may affect the immune response
	Severe infection/hematologic disease prior to treatment	Patients with severe infection or hematological diseases such as leukemia or lymphoma within 4 weeks before ICIs initiation
	ICIs allergy or contraindication	There is a clear history of drug allergy, autoimmune disease activity and other contraindications to ICIs use
	Severe heart, liver, kidney and other basic diseases	Severe cardiac insufficiency, liver and kidney failure, etc., unable to tolerate ≥ 2 cycles of systematic anti-tumor therapy

### 2.2 Treatment protocol

All patients received either ICIs monotherapy or ICIs combination therapy with chemotherapy. (1) ICIs combination chemotherapy regimens: Pembrolizumab (Merck Sharp & Dohme, National Drug Approval No. S20180019) 200 mg intravenously on day 1, plus cisplatin (Jiangsu Hansoh Pharmaceutical Group Co., Ltd., National Drug Approval No. H20040813) 80 mg/m² on day 1, and fluorouracil (Hainan Zhotai Pharmaceutical Co., Ltd., National Drug Approval No. H20051627) 800 mg/m² on days 1-5; or camrelizumab (Suzhou Shengdiya Biomedical Co., Ltd., National Drug Approval No. S20190027) 200 mg intravenously on day 1, combined with cisplatin 75 mg/m² on day 1 and paclitaxel (Yangtze River Pharmaceutical Group, National Drug Approval No. H20053001) 175 mg/m² on day 1. All treatments were administered intravenously in 21-day cycles. (2) ICIs monotherapy regimens: Pembrolizumab 200 mg intravenously every 21 days, or camrelizumab 200 mg intravenously every 14 days. Among the patients, 80.89% (127/157) with advanced ESCC received first-line therapy, while 19.11% (30/157) underwent second-line or subsequent therapies. In terms of treatment regimens, 67.52% (106/157) were treated with ICIs monotherapy, and 32.48% (51/157) received ICIs combination chemotherapy.

### 2.3 Data collection

The laboratory test data and matched clinical information of patients prior to their initial ICIs treatment were collected through the electronic medical record system. These data included gender, age, histological differentiation grade, tumor location, tumor size, smoking history, alcohol consumption history, pre-existing comorbidities, serum albumin levels, and lymphocyte counts. The PNI was calculated using the following formula: $PNI\;=\;serum\;albumin\;\left(g/dL\right)\;+\;5\;\times \;lymphocyte\;count\;(\times 10^{9}/L)$.

To ensure data accuracy, a dual-independent data entry and cross-verification mechanism was implemented, followed by logical checks and outlier validation. Cases with missing key indicators or inconsistent records were excluded. After all data were verified as correct, the database was locked to minimize data bias inherent in retrospective studies.

### 2.4 Clinical efficacy evaluation and follow-up

Treatment response was evaluated according to RECIST 1.1 (9), including complete response (CR), partial response (PR), stable disease (SD), and progressive disease (PD). All patients were followed up via outpatient visits or telephone interviews. The study period started from the date of the first immunotherapy session, with a minimum follow-up of 3 years until June 30, 2024. Patients were assessed every 6–8 weeks. The study endpoints were patient death or the end of follow-up. Progression-free survival (PFS) and overall survival (OS) were calculated in months. PFS was defined as the time from initiation of ICIs therapy to disease progression or death from any cause. OS was defined as the time from treatment initiation to death from any cause.

### 2.5 Statistical analysis

All data were analyzed using SPSS 26.0 software. Normally distributed measurement data were expressed as mean ± standard deviation (mean ± SD) and analyzed using the t-test. Non-normally distributed data were described as median (IQR) and analyzed using the Kruskal-Wallis H test. Categorical data were presented as [n (%)] and compared using the *χ2* test. The predictive value of the PNI for disease progression in patients with advanced ESCC was evaluated using receiver operating characteristic (ROC) curve analysis. The optimal cutoff value was determined by maximizing the Youden index. Kaplan-Meier method was used to construct survival curves for advanced ESCC patients in the high-PNI and low-PNI groups, and the log-rank test was used to compare differences between the two groups. Cox regression analysis was used to identify factors influencing the survival of advanced ESCC patients. Variables with a p-value< 0.10 in univariate analysis were entered into a multivariate Cox regression model to control for confounding factors and identify independent prognostic factors. Multicollinearity was assessed using the variance inflation factor (VIF), with a VIF > 10 indicating significant multicollinearity. A *p*< 0.05 was considered statistically significant.

## 3 Results

### 3.1 Analysis of ICIs efficacy in advanced ESCC patients

Among the 157 patients with advanced ESCC, the CR rate was 0.64% (1/157), the PR rate was 47.13% (74/157), the SD rate was 38.22% (60/157), and the PD rate was 14.01% (22/157). The median PFS was 14.2 months. During the follow-up period, a total of 80 patients died, resulting in a mortality rate of 50.96%.

### 3.2 Comparison of albumin, lymphocyte count, and PNI in patients with different therapeutic effects

**Figure 2:**
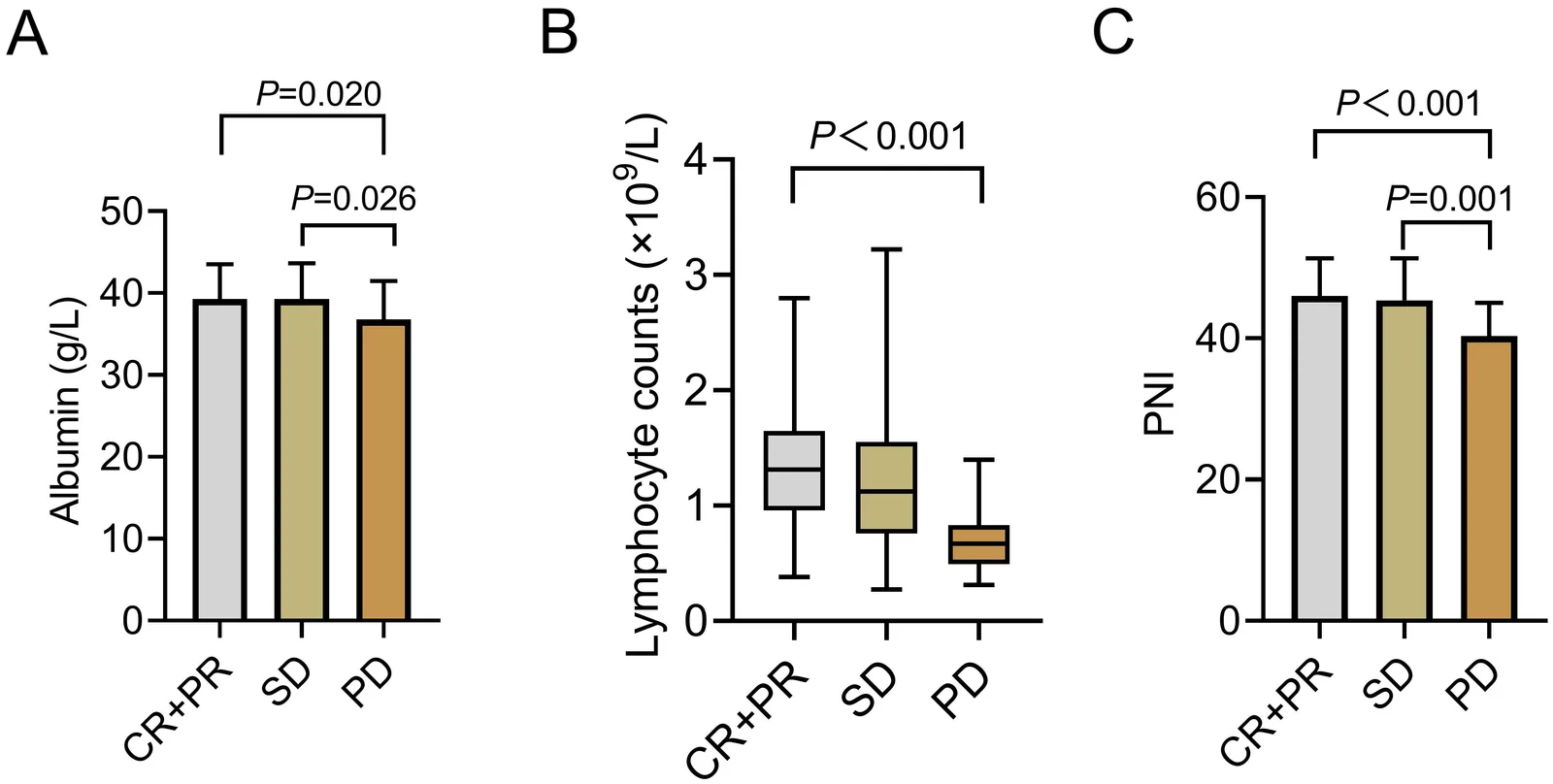
Comparison of albumin, lymphocytes, and PNI in patients with different therapeutic effects. **(A)** Albumin; **(B)** Lymphocytes; **(C)** PNI. Boxes represent the median (interquartile range, IQR); horizontal lines within boxes indicate the median; whiskers extend to the maximum and minimum values (within 1.5 × IQR). CR, complete response; PR, partial response; SD, stable disease; PD, progressive disease; PNI, prognostic nutritional index.

Statistically significant differences were observed among the three patient groups in terms of albumin levels, lymphocyte counts, and the PNI (*p*< 0.05, **Figure 2**). Specifically, both the CR+PR group and the SD group exhibited significantly higher albumin levels, lymphocyte counts, and PNI compared to the PD group (*p*< 0.05, **Figure 2**).

### 2.3 The evaluation value of PNI for disease progression in patients with advanced ESCC

**Figure 3:**
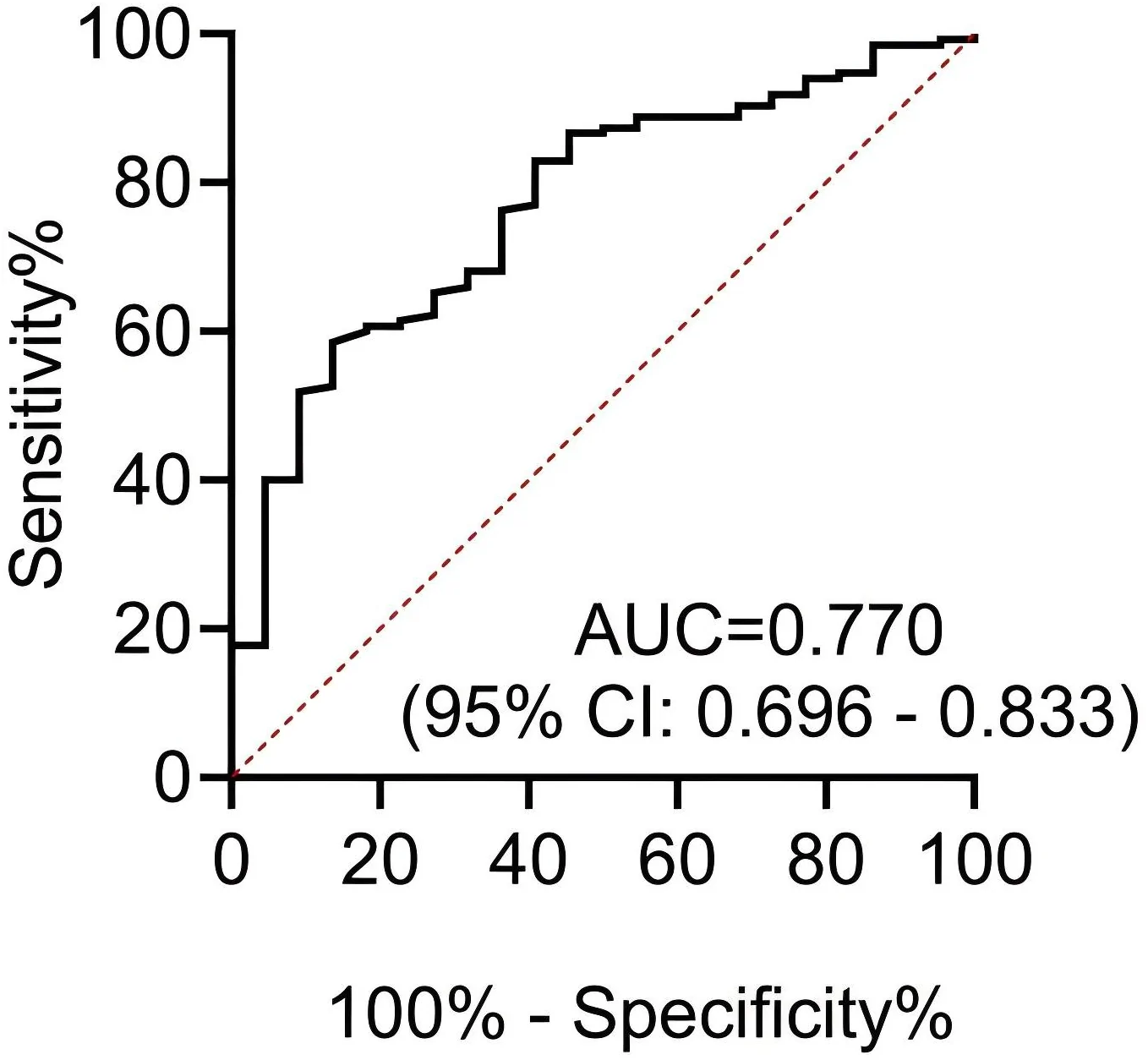
ROC curve for evaluating disease progression in patients with advanced ESCC using PNI. AUC, area under the curve; PNI, prognostic nutritional index.

According to the efficacy evaluation criteria, patients who experienced PD during the follow-up period were classified into the progression group (n=22), while those with CR, PR, or SD were assigned to the non-progression group (n=135). The optimal cutoff value for the PNI was determined by maximizing the Youden index. Results showed that the AUC of the PNI for predicting disease progression was 0.770, with an optimal cutoff value of 44.4, corresponding to a sensitivity of 86.36% and a specificity of 58.52% (**Figure 3**). The clinical implication of this cutoff is that patients with a PNI ≤ 44.4 are classified as the “high progression risk group”, indicating poor nutritional-immunity status and a higher likelihood of disease progression, thereby facilitating early identification of patients requiring intensified supportive care.

### 3.4 The relationship between PNI and clinical characteristics of advanced ESCC patients

Based on the optimal cutoff value, patients were categorized into a high-PNI group (PNI > 44.4, n=82) and a low-PNI group (PNI ≤ 44.4, n=75). Compared with the low-PNI group, the high-PNI group exhibited a significantly lower proportion of disease progression (*p*< 0.05, **Table 2**). There were no statistically significant differences between the two groups in terms of age, sex, smoking history, tumor location, degree of differentiation, or comorbid underlying diseases (*p*< 0.05, **Table 2**).

**Table 2:** The relationship between PNI and clinical characteristics of ESCC patients.

Parameter	Low-PNI (n=75)	High-PNI (n=82)		*p*
Sex [n (%)]			0.223	0.637
male	60 (80.00)	68 (82.93)		
female	15 (20.00)	14 (17.07)		
Age [n (%)]			0.842	0.359
Over 65 years old	51 (68.00)	50 (60.98)		
≤ 65 years old	24 (32.00)	32 (39.02)		
Smoking history [n (%)]	33 (44.00)	41 (50.00)	0.566	0.452
Drinking history [n (%)]	23 (30.67)	29 (35.37)	0.391	0.532
Tumor location [n (%)]			2.367	0.124
Upper thoracic segment	8 (10.67)	16 (19.51)		
Mid chest/lower segment	67 (89.33)	66 (80.49)		
Differentiation degree [n (%)]			0.100	0.752
Poorly differentiated	56 (74.67)	63 (76.83)		
Medium/high differentiation	19 (25.33)	19 (23.17)		
Combined radiotherapy [n (%)]	27 (36.00)	24 (29.27)	0.809	0.368
Immunotherapy line			1.176	0.278
first-tier	58 (77.33)	69 (84.15)		
Second tier and above	17 (22.67)	13 (15.85)		
Combined underlying diseases [n (%)]				
diabetes	9 (12.00)	11 (13.41)	0.071	0.791
hypertension	22 (29.33)	34 (41.46)	2.512	0.113
Tumor size [n (%)]			0.943	0.331
≥4cm	46 (61.33)	44 (53.66)		
<4cm	29 (38.67)	38 (46.34)		
Disease progression [n (%)]	19 (25.33)	3 (3.66)	15.273	<0.001

PNI, prognostic nutritional index.

### 3.5 Comparison of survival status between high-PNI group and low-PNI group patients

**Figure 4:**
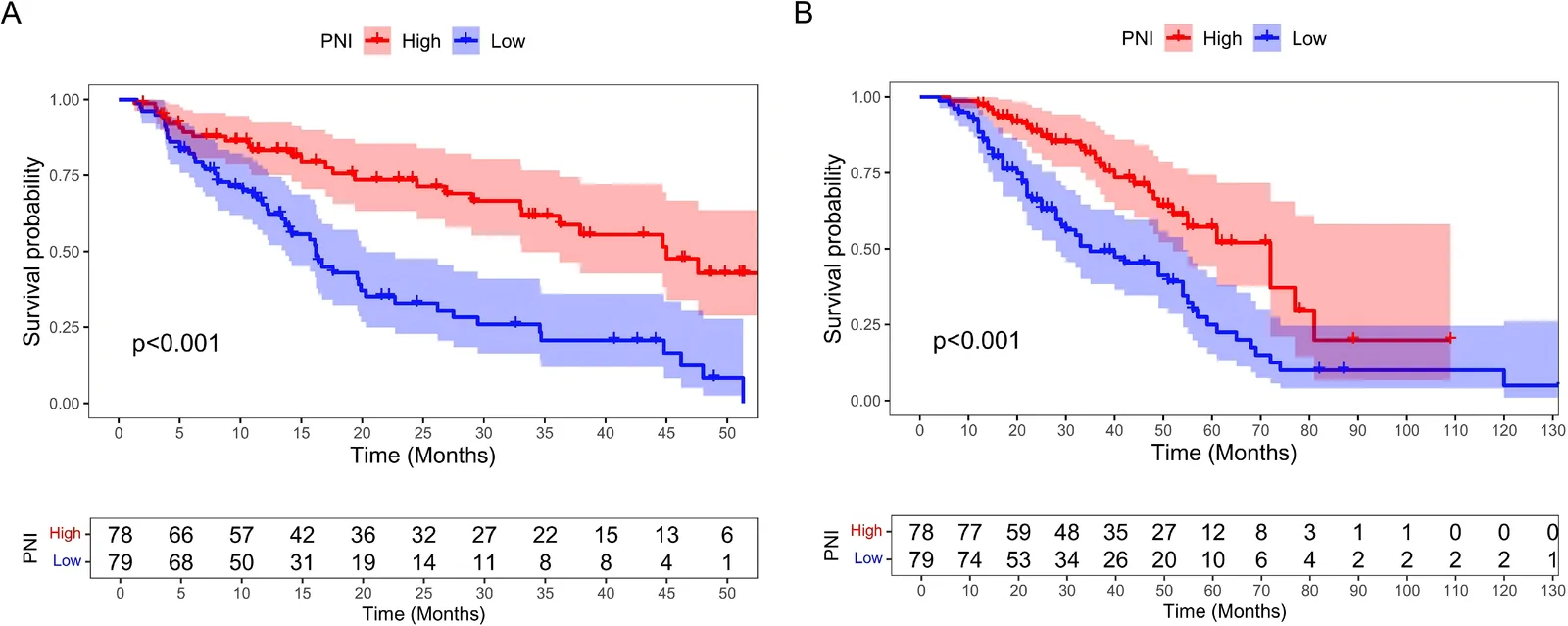
Survival analysis curves of advanced ESCC patients with different PNI levels. **(A)** Survival analysis curves of advanced ESCC patients for PFS; **(B)** Survival analysis curves of advanced ESCC patients for OS. Based on the optimal cutoff value (44.4) determined by the Youden index from ROC curve analysis, patients were categorized into a high PNI group (>44.4, n=82) and a low PNI group (≤44.4, n=75). Survival rates were estimated using the Kaplan–Meier method, and differences between groups were compared using the two-sided log-rank test. PNI, prognostic nutritional index. OS, overall survival; PFS, progression-free survival.

The median follow-up duration for this study was 48.5 months. All 157 patients were included in the survival analysis during the follow-up period. Kaplan-Meier analysis demonstrated that advanced ESCC patients in the high-PNI group exhibited significantly better PFS and OS compared to the low-PNI group (median PFS: 15.6 months vs 13.4 months, *p*< 0.001; median OS: 37.2 months vs 25.6 months, *p*< 0.05). The disease progression-free rate post-ICIs was 96.34% in the high-PNI group, substantially higher than the 74.67% observed in the low-PNI group (Log-rank χ² = 18.332, *p*< 0.05, **Figure 4A**). Similarly, the 3-year survival rate after ICIs treatment was 64.93% for high-PNI patients versus 33.75% for low-PNI patients (Log-rank χ² = 12.378, *p*< 0.05, **Figure 4B**).

### 3.6 Univariate and multivariate analysis of prognostic factors affecting patients with advanced ESCC

Then, univariate and multivariate Cox regression analyses were performed with PFS and OS as the outcome variables, respectively.

In the univariate analysis, albumin, lymphocyte count, and PNI were significantly associated with PFS in patients with advanced ESCC (P<0.001, **Table 3**). Since PNI is a composite nutritional index calculated based on albumin and lymphocyte count, substantial mathematical collinearity exists among these three variables. Collinearity diagnostics revealed a tolerance value of 0.000 for PNI, indicating severe collinearity between PNI and albumin/lymphocyte count. Therefore, based on the results of the univariate analysis, PNI and immunotherapy line were included in the multivariate Cox regression model for adjustment. The results demonstrated that after adjusting for immunotherapy line, PNI remained an independent protective prognostic factor for PFS in patients with advanced ESCC (HR: 0.876, 95% CI: 0.837–0.917, P<0.05, **Table 3**), whereas first-line immunotherapy was not an independent predictor of PFS (P>0.05, **Table 3**).

**Table 3:** Univariate and multivariate analysis of PFS in patients with advanced ESCC.

Clinical parameters	Univariate		Multivariate	
	*HR* (95%*CI*)	*p*	*HR* (95%*CI*)	*p*
Gender (Male)	0.871 (0.506~1.499)	0.622	—	—
Age (>60 years old)	1.084 (0.683~1.719)	0.734	—	—
Immunotherapy line (first-line)	0.638 (0.387~1.050)	0.077	0.901 (0.540~1.505)	0.691
Degree of differentiation (high differentiation)	1.240 (0.732~2.100)	0.424	—	—
Tumor location (upper chest)	1.098 (0.625~1.927)	0.746	—	—
Tumor size (≥ 4cm)	1.119 (0.717~1.748)	0.620	—	—
Smoking history (Yes)	0.934 (0.600~1.454)	0.763	—	—
Drinking history (yes)	1.052 (0.662~1.673)	0.830	—	—
Hypertension (Yes)	0.807 (0.514~1.267)	0.352	—	—
Diabetes (Yes)	1.178 (0.621~2.235)	0.615	—	—
Combined radiotherapy (Yes)	1.322 (0.843~2.075)	0.224	—	—
albumin	0.887 (0.843~0.934)	<0.001	—	—
Lymphocyte count	0.274 (0.160~0.471)	<0.001	—	—
PNI	0.874 (0.836~0.914)	<0.001	0.876 (0.837~0.917)	<0.001

PNI, prognostic nutritional index; HR, hazard ratio; CI, confidence interval.

Moreover, the univariate analysis showed that poor differentiation, smoking history, albumin, lymphocyte count, and PNI were significantly associated with OS (P<0.05, **Table 4**). Following the modeling strategy used in the PFS analysis, only poor differentiation, smoking history, and PNI were included in the multivariate Cox regression model. The results indicated that poor differentiation (HR: 2.033, 95% CI: 1.131–2.968), smoking history (HR: 1.832, 95% CI: 1.131–2.968), and PNI (HR: 0.909, 95% CI: 0.872–0.947) were independent prognostic factors for OS in patients with advanced ESCC (P<0.05, **Table 4**).

**Table 4:** Univariate and multivariate analysis of OS in patients with advanced ESCC.

Clinical parameters	Univariate		Multivariate	
	*HR* (95%*CI*)	*p*	*HR* (95%*CI*)	*p*
Gender (Male)	1.408 (0.804~2.467)	0.232	—	—
Age (>60 years old)	1.021 (0.643~1.621)	0.928	—	—
Immunotherapy line (first-line)	1.322 (0.791~2.208)	0.287	—	—
Degree of differentiation (high differentiation)	1.752 (1.023~2.999)	0.041	2.033 (1.131~2.968)	0.014
Tumor location (upper chest)	1.084 (0.615~1.908)	0.781	—	—
Tumor size (≥ 4cm)	0.661 (0.419~1.042)	0.075	—	—
Smoking history (Yes)	1.725 (1.084~2.744)	0.021	1.832 (1.131~2.968)	0.014
Drinking history (yes)	1.557 (0.974~2.488)	0.064	—	—
Hypertension (Yes)	0.996 (0.632~1.568)	0.985	—	—
Diabetes (Yes)	0.803 (0.423~1.525)	0.503	—	—
Combined radiotherapy (Yes)	0.722 (0.445~1.173)	0.189	—	—
albumin	0.936 (0.893~0.981)	0.006	—	—
Lymphocyte count	0.470 (0.289~0.764)	0.002	—	—
PNI	0.927 (0.892~0.964)	< 0.001	0.909 (0.872~0.947)	< 0.001

PNI, prognostic nutritional index; HR, hazard ratio; CI, confidence interval.

## 4 Discussion

In recent years, ICIs have demonstrated remarkable efficacy in treating various cancers. By blocking immune checkpoints, ICIs reactivate the body’s immune system, enabling it to recognize and eliminate tumor cells, thus providing a novel therapeutic option for patients (10, 11). However, significant heterogeneity exists in patient responses to ICIs therapy, with some achieving long-term survival while others derive little clinical benefit. Therefore, accurate prognostic assessment has important implications for patients with advanced ESCC receiving ICIs therapy.

Recently, the PNI has gained increasing attention in the field of tumor prognosis assessment. Calculated by integrating serum albumin levels and peripheral blood lymphocyte counts, PNI offers unique advantages in reflecting patients’ nutritional and immune status, thereby providing valuable references for clinical decision-making (12, 13). The results of this study demonstrated that patients in the response and SD groups exhibited significantly higher albumin levels, lymphocyte counts, and PNI values than those in the PD group. The AUC of PNI for predicting disease progression reached 0.770. Furthermore, Cox multivariate analysis identified PNI as an independent prognostic factor affecting both PFS and OS in patients with advanced ESCC. These findings suggested that PNI holds significant value in the prognostic evaluation for advanced ESCC patients undergoing ICIs therapy. From a nutritional perspective, a higher PNI reflects better nutritional status in patients. As a key component of PNI calculation, serum albumin levels indicate whether patients have adequate protein intake and the ability to synthesize proteins to maintain normal physiological functions and metabolic demands (14). In cancer patients, optimal nutritional status helps enhance energy reserves and improve tissue repair and regenerative capacity. Adequate protein availability serves as the essential substrate for the production and function of immune cells, thereby ensuring proper immune system operation (15). Patients with good nutritional status generally exhibit better tolerance to ICIs therapy, enabling them to withstand treatment-related adverse effects more effectively and ensuring treatment continuity. Conversely, low PNI values may indicate malnutrition, which can result in metabolic dysregulation, impaired immune function, and consequently compromise the efficacy of ICIs treatment, leading to poor patient prognosis (16). From an immunological perspective, the lymphocyte count used in the PNI serves as a crucial indicator of the body’s immune function. Patients in the high-PNI group exhibit elevated lymphocyte counts, suggesting relatively stronger immune function. Lymphocytes play a pivotal role in antitumor immunity: T lymphocytes directly eliminate tumor cells by releasing cytotoxic substances such as perforin and granzymes, thereby inducing tumor cell apoptosis (17, 18). Additionally, they secrete cytokines to modulate immune responses, activate other immune cells, and enhance the body’s antitumor immunity (19). As reported by Zhang X et al. (20), peripheral blood lymphocyte levels are associated with prognosis in various cancers, including gastric, liver, and esophageal cancers. B lymphocytes produce antibodies that contribute to tumor cell clearance, while natural killer (NK) cells mediate nonspecific tumor cell lysis. An optimal balance of peripheral immune cells correlates with improved patient outcomes. During ICIs therapy, activation of the immune system is essential for therapeutic efficacy. Patients with normal or robust immune function have a greater ability to recognize and respond to ICIs (21), thereby reactivating immune surveillance and tumor-killing mechanisms, ultimately improving treatment outcomes and prolonging survival. Therefore, monitoring PNI values enables timely assessment of a patient’s condition, providing clinicians with critical insights for prognostic evaluation and personalized treatment strategies. This study found that the optimal PNI cutoff value (44.4) achieved a high sensitivity (86.36%) while maintaining a specificity of 58.52%, suggesting that it is more suitable as a screening tool than a diagnostic tool. Specifically, patients with a PNI ≤ 44.4 should be considered at high risk for disease progression, and enhanced imaging follow-up or nutritional intervention is recommended for this population.

In addition to the PNI, this study identified poor differentiation and smoking history as independent factors influencing the prognosis of advanced ESCC patients undergoing ICIs therapy. The degree of tumor differentiation serves as a critical indicator of tumor cell malignancy. Poorly differentiated ESCC cells exhibit greater invasiveness and metastatic potential. They show significant morphological and structural deviations from normal tissue cells, along with rapid proliferation, local invasion, and a propensity for distant metastasis (22). During ICIs treatment, poorly differentiated tumor cells may evade immune system attacks through various mechanisms, such as downregulating tumor-associated antigen expression or secreting immunosuppressive factors, thereby compromising ICIs efficacy and shortening patient survival (23). Smoking is a major risk factor for ESCC. Chronic smoking causes persistent stimulation and damage to the esophageal mucosa via harmful substances, triggering a cascade of molecular biological alterations that increase the risk of ESCC development. For patients already diagnosed with ESCC and receiving ICIs therapy, smoking further impairs immune function (24), consequently reducing ICIs effectiveness and adversely affecting prognosis. In clinical prognostic assessments, the aforementioned factors should be evaluated comprehensively to guide tailored therapeutic strategies, such as interventions for smoking cessation, aimed at enhancing treatment outcomes.

These findings hold significant clinical implications. In terms of patient prognosis assessment, the PNI serves as a simple, convenient, and cost-effective indicator, providing clinicians with a novel evaluation tool. For patients with lower PNI values, poorly differentiated tumors, or a history of smoking—factors associated with unfavorable prognosis—clinicians may consider adopting more intensive therapeutic strategies, such as combined radiotherapy or other emerging treatment approaches. These patients may potentially derive greater clinical benefit from such interventions. However, as this study was based on a retrospective design, the observed association between PNI and survival outcomes does not establish a causal relationship. Therefore, these clinical implications require further validation in prospective studies.

However, this study has several limitations. First, the sample size was relatively small, with only 157 patients with advanced ESCC included, and the number of progression events was limited (n=22), which may have affected the statistical stability and generalizability of the optimal cutoff value determined by ROC curve analysis. Therefore, the proposed PNI cutoff value (44.4) should be regarded as an exploratory finding and requires further validation in larger, multicenter prospective cohorts. Second, due to the limitations of data completeness inherent to the retrospective study design, several important potential confounding factors that have been demonstrated to be closely associated with the prognosis of ICI therapy in ESCC were not included in the multivariate analysis, including ECOG performance status, tumor metastatic burden, and PD-L1 expression status. These variables may substantially influence the efficacy of ICI therapy and survival outcomes in patients with advanced ESCC and could potentially confound the association between PNI and PFS. Future studies should explore the integration of PNI with other clinical and molecular parameters to establish more comprehensive and accurate prognostic prediction models. A multidimensional assessment incorporating multiple indicators may improve the accuracy of prognostic evaluation for ICI-treated patients with advanced ESCC and provide stronger evidence to support clinical decision-making. Third, ROC curve analysis for predicting disease progression demonstrated a specificity of only 58.52%, suggesting that the discriminatory ability of PNI as an independent prognostic stratification tool remains suboptimal. Therefore, in clinical practice, PNI should be combined with other clinical parameters for comprehensive risk assessment. Fourth, the optimal PNI cutoff value identified in this study was derived from a single-center dataset, and no independent external validation cohort was available. The robustness and generalizability of this cutoff value require confirmation in larger-scale, multicenter prospective cohorts.

In conclusion, PNI is associated with disease progression after ICI therapy in patients with advanced esophageal squamous cell carcinoma and may serve as a promising adjunctive biomarker for prognostic assessment in this population.

## Data Availability

The original contributions presented in the study are included in the article/supplementary material. Further inquiries can be directed to the corresponding author.
